# Limited evidence for return to sport testing after ACL reconstruction in children and adolescents under 16 years: a scoping review

**DOI:** 10.1186/s40634-020-00298-8

**Published:** 2020-10-15

**Authors:** Martijn Dietvorst, Maarten H. Brzoskowski, Marieke van der Steen, Eugenie Delvaux, Rob P. A. Janssen, Nicky Van Melick

**Affiliations:** 1 Department of Orthopaedic Surgery, Máxima MC, Eindhoven, the Netherlands; 2 Fontys University of Applied Sciences, Eindhoven, the Netherlands; 3 Department of Orthopaedic Surgery, Catharina Hospital Eindhoven, Eindhoven, the Netherlands; 4 Medical Library, Máxima MC, Veldhoven, the Netherlands; 5 Orthopaedic Biomechanics, Department of Biomedical Engineering, Eindhoven University of Technology, Eindhoven, the Netherlands; 6 Knee Expert Center Eindhoven, St. Anna hospital Geldrop, Geldrop, the Netherlands

**Keywords:** “anterior cruciate ligament”, “Paediatric”, “Adolescent”, “return to sport”, “Scoping review”

## Abstract

Specific return to sport criteria for children and adolescents after anterior cruciate ligament injury and reconstruction are unknown. The aim of this scoping review is to provide an overview of current tests regarding return to sport for children and adolescents. This scoping review was performed according to the PRISMA statement. A systematic search was performed on PubMed and EMBASE. The inclusion criteria were diagnostic and prognostic studies evaluating tests regarding return to sport after ACL injury and reconstruction in children/adolescents (age < 18 years). Twenty-six studies were included, of which 22 studies evaluated tests in the age category of 16 to 18 years. All studies evaluated tests after ACL reconstruction, no studies have been conducted in non-operative patients. Strength tests, movement quality and patient reported outcomes measures (PROMs) are investigated most frequently. Clearance for return to sport should be based on a test battery including strength tests, movement quality during sport-specific tasks and (paediatric) patient reported outcome measures. There are no recommendations on which specific tests regarding quantity and quality of movement should be used. Future research should aim at at developing and validating a test battery including movement quality and neuromotor control in a sport-specific context for both younger children and adolescents after both operative and non-operative treatment.

## Introduction

Anterior cruciate ligament (ACL) ruptures in children and adolescents are considered to be a severe injury of the knee in a vulnerable population with high rates of secondary ruptures after ACL reconstruction [1]. There are two possible treatment options for children with an ACL rupture according to the International Olympic Committee (IOC): conservative high quality rehabilitation or surgical ACL reconstruction plus high quality rehabilitation [1, 25]. The goal of either treatment regimen is to restore a stable, well-functioning knee, to reduce the risk of further meniscal or chondral injury and to successfully return to sport [1]. Successful return to sport can be defined as returning to the desired level of sport without sustaining a second ACL injury.

The IOC statement recommends using functional performance tests and return to sport criteria during rehabilitation [1]. The specific clinical and functional milestones described in the four-phased rehabilitation are based on the outcomes of a systematic review and practice guideline by Van Melick et al. [40] This systematic review, however, excluded skeletally immature children and it is therefore unknown if these milestones can be applied in the younger population [40].

The aim of this scoping review is to provide an overview of the current evidence of tests evaluating readiness for return to sport after ACL injury or ACL reconstruction in children and adolescents (age < 18 years). Based on the outcomes of this scoping review, the hiatus in the current evidence is shown and advice is given for future research.

## Methods

This scoping review was performed following the Preferred Reporting Items for Systematic Reviews and Meta-Analysis (PRISMA) statement extension for scoping reviews [37]. The general purpose for inducting a scoping review is to identify and map the available evidence and not to produce a critically appraised and synthesised answer to a specific question [26].

### Selection criteria

Articles included in the current scoping review had to meet the inclusion and exclusion criteria listed in Table 1.

**Table 1:** Overview of inclusion and exclusion criteria for this scoping review

	Inclusion criteria	Exclusion criteria
**Participants**	Children (average age < 18 years)	Average age ≥ 18 years
**Injury**	ACL rupture or reconstruction	ACL revision surgery
		Multi-ligament injury of the knee
		Fractures
**Tests**	Any test concerning return to sport, including:	
	- Strength tests	
	- Hop tests	
	- Movement quality tests	
	- Physical examination	
	- PROMs	
**Outcomes**	Diagnostic values (e.g.,sensitivity, specificity)	
	Prognostic information (e.g.,correlation coefficients, regression)	
**Study design**	Cross-sectional studies	
	Longitudinal studies	

### Search strategy

At the 30th of March 2020, an information specialist (ED) performed a systematic literature search in PubMed (Medline) and EMBASE databases, as shown in Additional file 1. All published articles up to the 30th of March of 2020 were considered eligible. The following terms, including synonyms and closely related words, were used as index terms or free-text words: “anterior cruciate ligament injury”, “paediatric”, “adolescent” and “return to sport”. Studies written in other languages than English, Dutch and German were excluded. Duplicate articles were removed.

### Study selection

Two researchers (MD, MB) independently screened the abstracts for eligibility by using the Rayyan QCRI app (rayyan.qrci.org) [28]. A full-text version of all eligible studies was reviewed. All references of these studies were screened for additional eligible articles. Any disagreements between the reviewers were resolved by discussion. Cohen’s kappa was calculated to measure inter-reviewer agreement in the selection process.

### Data collection process

Two authors (MD, MB) extracted all relevant data. The data included specific details of the tests, population characteristics, interventions, study methods, follow-up period and outcomes of interest to the review question and research objectives. Any disagreements about the interpretation of the results were resolved by discussion. Due to the heterogeinity of the study designs and data and the aim of this scoping review, no risk of bias assessment was performed on the included studies.

## Results

### Search results

**Fig. 1:**
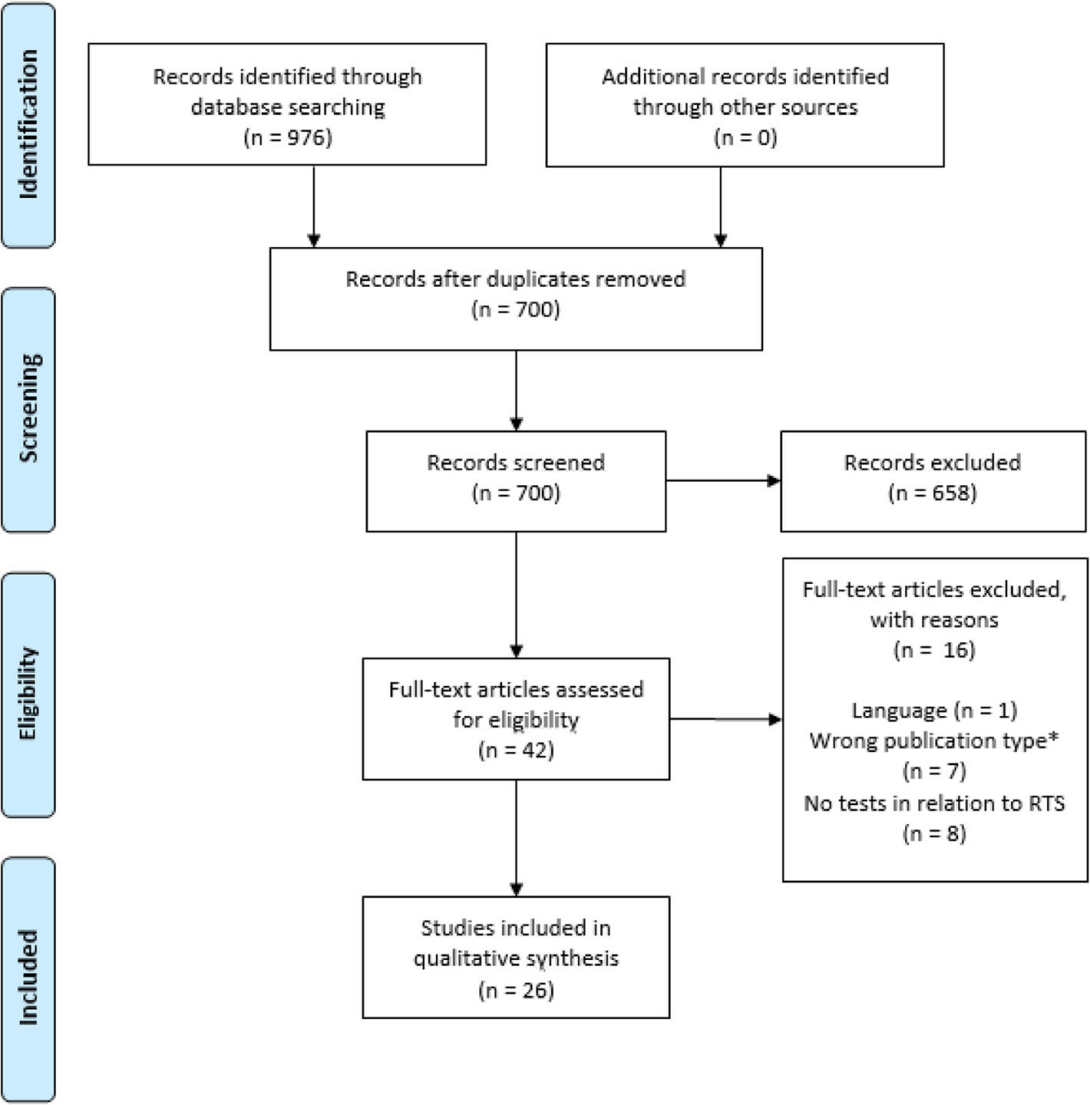
PRISMA flow diagram of inclusion process. *including abstracts of presentations

Twenty six studies were included in this scoping review (Fig. 1). The inter-reviewer agreement was almost perfect with a Cohen’s Kappa of 0.94. The 3 studies of conflict were resolved by discussion. All 26 studies are published in the last 10 years and 22 in the last 5 years.

### Study characteristics

Fifteen studies had a cross-sectional design [4, 7, 9, 11, 14, 16, 18, 21, 27, 29, 32, 34, 35, 43, 44]. Eleven studies had a longitudinal design [3, 10, 12, 19, 20, 22, 23, 30, 31, 33, 36]. Sixteen of the 26 included studies were from the same research group from Cincinnati Children’s Hospital Medical Center [11, 14, 18–22, 27, 30–33, 35, 36, 44]. Ten of those sixteen studies reported to be part of a larger, prospective study on ACL reconstruction outcomes (ACL-RELAY study) [11, 18–20, 22, 30, 34–36, 44].

### Demographic characteristics

**Fig. 2:**
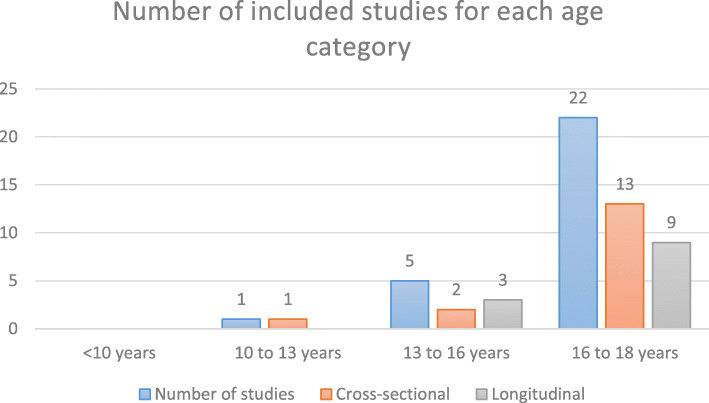
Number (*n* = 26) of included studies for each mean age divided in categories. Two studies investigated two age categories which were presented seperately [7, 18].

The exact number of included patients in this scoping review is difficult to estimate due to that some studies include participants from the same prospective study [11, 18–20, 22, 30, 34–36, 44]. The range of included patients are 14 to 384 [10, 29]. The number of patients for each study are shown in Additional file 2. The majority of the studies included participants of 16 to 18 years of age [4, 7, 10, 11, 14, 16, 18–23, 27, 29–36, 44]. Six studies included children younger than 16 years of age [3, 7, 12, 18, 31, 33]. Fig. 2 shows the number of studies for each age category.

### Surgical procedures

All studies investigated patients after ACL reconstruction (ACLR). No study regarding return to sport testing after non-operative treatment were available for inclusion. Four studies evaluated tests in children who had undergone a physeal sparing or transphyseal procedure, as is shown in Table 2 [3, 7, 12, 18]. To reconstruct the ACL, a hamstring tendon autograft was used most frequently (*n* = 18), followed by patella tendon autograft (*n* = 16).

**Table 2:** Overview of studies providing data on modifications of ACLR due to open physes

Studies	Procedure type
Astur [3]	Transphyseal
Boyle [7]	Transphyseal
Dekker [12]	Transphyseal, all-epiphyseal and partial-transphyseal
Ithurburn [18]	All-epiphyseal

### Tests regarding return to sport

#### Muscle strength tests

Thirteen of the 26 studies investigated the outcomes of muscle strength tests in relation to outcomes regarding return to sport [4, 9, 11, 14, 16, 18, 19, 21, 22, 29, 34–36]. In Additional file 2, the different muscle strength tests are shown for each study in Table 1. In all 13 studies quadriceps strength was evaluated. Isometric quadriceps strength was evaluated in 8 studies [4, 9, 14, 19, 21, 22, 34, 35]. Eight studies included isokinetic quadriceps strength tests [4, 9, 11, 16, 18, 22, 29, 36]. Hamstring strength was also tested in 8 studies, of which 2 studies evaluated isometric hamstring strength [4, 9] and 8 studies isokinetic hamstring strength [4, 9, 11, 16, 18, 22, 29, 36]. Hip abduction strength was tested in 4 studies [11, 14, 16, 22] and hip external rotation strength in one study [16]. Most studies evaluated the strength tests at return to sport (RTS) around 8 months post-ACLR. Six studies evaluated strength tests as a prognostic value for a variety of outcomes, such as movement quality outcomes, PROMs, re-ruptures and achieving RTS at follow up [9, 19, 21, 22, 29, 36]. One study evaluated the prognostic value of strength tests for achieving RTS and one study for sustaining an ipsilateral re-rupture [9, 29].

#### Hop tests

Four studies [4, 18, 36, 43] evaluated hop tests in regard to RTS, of which two studies [18, 36] tested Noyes’ hop test battery -single hop for distance, triple hop for distance, crossover hop and 6-m timed hop-. One study tested a single hop for distance [43]. One study analysed the hop test for distance, a vertical hop test and side hop test (see Additional file 2, Table 2) [4]. Testing occurred in two studies at RTS (around 8 months) and in one study before RTS (7 months). Toole et al. evaluated Noyes’ hop test battery as a prognostic value for achieving combined test crition cut-offs after 1 year follow-up post-RTS [36].

#### Movement quality

In 14 studies movement quality in relation to RTS was evaluated with a great variety of different parameters, as is shown in Additional file 2, Table 3 [7, 10, 11, 14, 16, 20, 21, 27, 29, 31–34, 43]. Eight studies tested different biomechanical variables during a landing task [14, 16, 20, 21, 27, 33, 34, 43]. In 11 of the 14 studies, testing was done at RTS (approximately 7 months post-ACLR) [7, 11, 14, 16, 20, 21, 29, 31–34]. Five studies evaluated movement quality as a prognostic factor after follow-up [10, 20, 29, 31, 33], of which four studies [10, 29, 31, 33] investigated movement quality as a prognostic value for sustaining re-ruptures and one study [20] as prognostic factor for outcomes of PROMs and hop tests.

#### Patient reported outcome measures

PROMs in relation to RTS were evaluated in 10 studies, as is shown in Additional file 2, Table 4 [3, 4, 9, 12, 18, 21, 23, 30, 36, 44]. The IKDC Subjective Knee Evaluation Form [21, 36, 44] and the ACL-RSI [4, 9, 23] are evaluated most frequently, followed by the Tegner Activity Scale [3, 4]. Of the paediatric PROMs, the Pedi-IKDC is tested in one study [9]. Seven studies evaluated PROMs at the moment of RTS (approximately 8 months post-ACLR) [3, 18, 21, 23, 30, 36, 44]. Prognostic values of PROMs were investigated in 7 studies [3, 9, 12, 21, 23, 30, 36], of which 4 studies [3, 12, 23, 30] tested the prognostic value of PROMs for developing a re-rupture, 2 studies [9, 12] for achieving RTS and one study for meeting combined test criterion cut-offs [36].

#### Physical examination

Outcomes of joint laxity tests and range of motion of joints in regard to RTS were investigated in respectively 3 [7, 22, 33] and 2 studies [16, 22], as shown in Additional file 2, Table 5. Laxity tests were performed with the KT-1000 arthrometer [7, 22, 33]. Two studies evaluated the prognostic values of laxity tests at the moment of RTS in relation to PROMs [22] and re-ruptures [33].

#### Test battery

Two studies tested the same test battery in relation to RTS, consisting of a combination of test criterion cut-off values of the IKDC, muscle strength LSI and hop tests LSI at RTS (see Additional file 2, Table 6) [18, 36]. Ithurburn et al. [18] analysed the proportions of participants meeting all RTS criterion cut-offs at RTS for each age category, while Toole et al. [36] analysed whether those proportions maintained the same level of sport participation after 1 year follow-up post-RTS.

### Return to sport clearance criteria

Seven studies included a definition of their RTS clearance criteria, including objective and subjective criteria [3, 7, 9, 10, 16, 23, 29]. All 7 studies used a combination of different tests to assess readiness for RTS. Table 3 provides an overview of tests for each study.

**Table 3:** Overview of tests used as RTS criteria. * including PROMs; ** including range of motion, effusion, laxity tests

Studies	Strength tests	Hop tests	Movement quality	Subjective outcomes*	Physical examination**	Time based
**Astur** [3]	X		X			
**Boyle** [7]	X		X	X	X	X
**Burland** [9]	X	X			X	
**Capin** [10]	X	X		X		
**Hannon** [16]	X		X		X	
**McPherson** [23]	X		X		X	
**Palmieri-Smith** [29]	X				X	

## Discussion

The most important finding of this scoping review was that many studies have evaluated strength tests, hop tests, movement quality and PROMs regarding return to sport in adolescents after ACL reconstruction, but that only few studies have been conducted in children/adolescents under 16 years of age. There is currently sparse evidence for specific testing regarding return to sport in younger children. However, in the category of 16 to 18 years many studies have been conducted, both comparing different tests at the moment of RTS as well as evaluating prognostic values of tests with regard to ACL graft rerupture, achieving return to preinjury sport level or subjective outcomes [3, 9, 10, 12, 19, 20, 22, 23, 29–31, 33, 36].

Successful return to sport is context- and outcome-dependent and has a different meaning for different people (including the patient, clinician and coach) [2]. Criteria of clearance RTS exist in great variability and it should be noted that a true clearance is multifactorial and complex [8]. This was reflected by the variability of tests and outcomes described in the included studies, including strength tests, hop tests, movement quality, PROMs and physical examination.

The 2018 IOC consensus statement on paediatric ACL recommends return to sport clearance criteria, including a LSI > 90% for strength and single-leg hop tests for adolescents, psychological factors, knowledge and gradual increase in sport specific training without pain and effusion [1]. All of the included studies which presented their RTS clearance criteria, used a combination of tests to determine whether the child or adolescent was ready to return to sport [3, 7, 9, 10, 16, 23, 29]. All of those studies used strength tests as a criterium [3, 7, 9, 10, 16, 23, 29]. Subjective outcomes and hop tests are used in only 2 studies respectively [7, 10]. One study used time as a criterium for RTS clearance, which is in contrast to the scoping review on RTS clearance after ACL reconstruction by Burgi et al. [7, 8] They found that 85% of the studies used time as the primary criterion to clear athletes (no age limits defined) to RTS [8]. Children and adolescents are at a higher risk of a second ACL injury, especially in the first year after ACL reconstruction [1]. It is therefore recommended to advise the child not to return to pivoting sport within 12 months after ACL reconstruction [1]. The timing of RTS testing in the included studies was approximately 7.5 months after ACL reconstruction, which seems to be early in this population. However, it is not known whether the child was allowed to return to pivoting sport.

Thirteen of the 26 included studies evaluated the outcomes of strength tests regarding to return to sport [4, 9, 11, 14, 16, 18, 19, 21, 22, 29, 34–36], of which one study compared strength tests in paediatric patients (mean 12 years of age) versus adolescents (mean 16.5 years of age) [18]. Besides, one study tested muscle strength in adolescents between 12 and 16 years of age [9]. All other studies evaluated strength tests in adolescents older than 16 years of age, which may resemble adults [1]. It is recommended that in the younger patients (< 12 years) less emphasis should be on muscle strength and hypertrophy [1]. Pre-pubertal children may benefit from resistance training, but the trainability of muscle strength increases with age [6]. During puberty, boys show an accelerated increase in muscle strength and girls continue to develop in a similar rate as pre-puberty [6]. Despite these gender-related differences in trainability and outcome, only one study evaluated the differences between males and females [36].

The included studies showed a great variety of measurements regarding the quantity of movement, including isokinetic and isometric strength tests and different hop tests. However, the LSI is often used as an outcome to describe symmetry during strength tests or hop tests. Caution must be taken when interpreting an LSI in absence of an accurate baseline measurement, which includes muscle strength LSI as well as hop tests LSI [5, 8, 38, 42, 43]. A normal LSI does not exclude postoperative deterioration of the uninvolved leg [8]. The IOC therefore recommends to focus on the quality of the movements during a single-leg hop test, instead of LSI [1]. Movement quality is the most frequently evaluated test category in the included studies, but also with a great variety of tests and outcomes [7, 10, 11, 14, 16, 20, 21, 27, 29, 31–34, 43]. Landing variables are evaluated most frequently and are advised to use as movement quality measurement. As ACL injuries are common in pivoting sport, stricter cut-offs for strength tests are recommended in case of a return to pivoting sport [40]. Furthermore, specific movement quality tests and outcomes might be relevant in return to pivoting sport because of the loss of normal knee proprioception, such as single leg movement including cutting mechanics. Based on a recent systematic review, RTS testing should include asymmetry in loading experienced by each limb rather than the movement patterns alone, as asymmetries between the limbs were more commonly identified in kinetic variables than in kinematic variables [17].

Besides strength tests, hop tests and movement quality, subjective outcomes such as PROMs might have an important role in determining readiness for return to sport. They offer a more complete picture of the patient’s perception on the actual recovery after ACL surgery [9]. Caution must be taken when interpreting PROMs scores in children and adolescents when adult PROMs are used in children instead of the specific paediatric PROMS due to problems in comprehensibility [13]. The Pedi-IKDC was described in one study, while the other studies used the (adult) IKDC and/or the KOOS [9, 18, 21, 36, 44]. Besides the IKDC and the KOOS, other PROMs are used and are not validated in children [9, 13, 30, 39]. This in accordance with the infrequent use of pediatric-specific instruments as outcomes measures in pediatric ACL literature [15]. The ACL-RSI is validated from the age of 16 years [41]. Specific paediatric versions of adult PROMS have been developed and should be used in evaluating children with knee injuries [13, 39]. One must note however, that in most of the included studies, the mean age is 17 years and that comprehensibility in that age category might not be a significant issue.

### Limitations

The most important limitation of this review is that data from sixteen of the 26 included studies are from the same research group and ten of those sixteen are from the same prospective cohort study (ACL RELAY) [11, 18–20, 22, 30, 34–36, 44]. It is therefore difficult to determine whether the same patients are evaluated in more than one study. It is important to note however, that these studies are published from a well-known high-quality American ACL research group and the use of measurements is based on their professional opinions and experiences. This adds to the value of the described tests in relation to return to sport.

Another important limitation of this study is that the majority of the included participants were older than 16 years of age and it may therefore be difficult to draw conclusions about return to sport criteria for younger children. This emphasizes the necessity to aim further research at younger children. Especially since the incidence of ACL injuries in this vulnerable group of is increasing [1].

### Recommendations for day-to-day practice

Clearance for RTS is a complex and multifactorial issue. The following recommendations for measurements in relation to RTS are made based on the results of this scoping review and expert opinions of the authors. It is important that rehabilitation must be guided by clinical and functional milestones as decribed in the IOC statement and to advise the child not to return to pivoting sports within 12 months after ACL reconstruction [1]. Tests regarding RTS clearance for adolescents (16–18 years old) should include quadriceps and hamstrings strength tests, hop tests, movement quality assessment during sport specific tasks and PROMs, which might in this age category be disputable whether paediatric of adult PROMs can be used. In the age category 12–16 years, testing should include hop tests, movement quality and paediatric PROMs [24]. Strengh tests in this age category are debatable as there is only sparse evidence of muscle strength tests and outcomes in this age category. In children younger than 12 years, there is currently very limited evidence and based on the physiological characteristics of this group, less emphasize should be on muscle strength and more on movement quality [1, 24]. In this age category, only paediatric PROMs are recommended to evaluate subjective outcomes [13]. Furthermore, it is important in all age categories to compare the postoperative values with preoperative test outcomes and/or reference values to assess postoperative deterioriation of muscle strength of the uninvolved limb. Normalized strength for body weight, compared to reference values, may also provide information about muscle strength [10].

### Recommendations for further research

Future research should aim at validating specific tests in children after ACL injury and after ACL reconstruction. Validation includes measuring reliability, validity and responsiveness, as these variables are unknown of many RTS tests [8]. There should be more focus on the movement quality as a test for RTS clearance, as altered neuromuscular function and biomechanics could be a risk factor for a second ACL rupture [40]. Besides, there are individual differences in neuromotor learning capacity and flexibility, this underlines the importance of the shift from time-based rehabilitation to a patientspecific goal-based rehabilitation [40]. The aim should be to develop a test battery measuring clinical outcomes, strength tests, hop tests, movement quality and PROMs based on a goal-based rehabilitation in a sport-specific context [8, 40]. As most of the studies evaluated tests in an adolescent population, we also recommend to aim future research at younger children (< 16 years of age) and to evaluate differences between the sexes. Since no studies have been conducted in non-operative patients, future research should also aim at this population. Tests regarding RTS after non-operative treatment may especially be relevant for skeletally immature children, as these children are often treated non-operatively [1].

## Conclusion

Many studies on tests regarding RTS have been conducted among adolescents after ACL reconstruction, while there are only few studies evaluating tests among younger children. Strength tests, movement quality and PROMs are most frequently evaluated and are useful to determine readiness for return to sport. Further research should aim at younger children and at developing and validating a test battery including movement quality and neuromotor control in a sport-specific context in both operative and non-operative patients.

## Supplementary information

**Additional file 1.**

**Additional file 2.**

## Abbreviations

ACL: Anterior cruciate ligament
ACLR: Anterior Cruciate ligament reconstruction
ACL RELAY: Anterior Cruciate Ligament REconstruction Long-term outcomes in Adolescents and Young adults
ACL-RSI: Anterior Cruciate Ligament - Return to Sport after Injury
(Pedi-)IKDC: (Paediatric) International Knee Documentation Committee Subjective Knee Form
IOC: International Olympic Committee
KOOS(−Child): Knee injury and Osteoarthritis Outcome Score (−Child)
KT-1000 Arthrometer: Knee laxity Testing device 1000 Arthrometer
LSI: Limb Symmetry Index
PRISMA: Preferred Reporting Items for Systematic Reviews and Meta-Analyses
PROMs: Patient Reported Outcomes Measures
RTS: Return To Sport
