# Supplementary material for: Limited evidence for return to sport testing after ACL reconstruction in children and adolescents under 16 years: a scoping review
Source: J Exp Orthop. 2020 Oct 15;7:83. doi: 10.1186/s40634-020-00298-8 (PMC7561621; doi:10.1186/s40634-020-00298-8)
Supplement: Supplementary file 1 — Additional file 1. [file 40634_2020_298_MOESM1_ESM.docx]

# Appendix 1 – search strategies

**Search date:** 30-3-2020

**Databases:** Pubmed and EMBASE

|  |
| --- |

***Results***

|  | **PUBMED** | **EMBASE** | **Total** |
| --- | --- | --- | --- |
|  | 547 | 429 | 976 |
| **After removing duplicates** | 285 | 415 | 700 |

***Search 30-03-2020***

| **Database** |  |
| --- | --- |
| Pubmed | - [AND in builder](https://www.ncbi.nlm.nih.gov/pubmed/advanced) - [OR in builder](https://www.ncbi.nlm.nih.gov/pubmed/advanced) - [NOT in builder](https://www.ncbi.nlm.nih.gov/pubmed/advanced) - [Delete from history](https://www.ncbi.nlm.nih.gov/pubmed/advanced) - [Show search results](https://www.ncbi.nlm.nih.gov/pubmed/advanced) - [Show search details](https://www.ncbi.nlm.nih.gov/pubmed/advanced) - [AND in builder](https://www.ncbi.nlm.nih.gov/pubmed/advanced) - [OR in builder](https://www.ncbi.nlm.nih.gov/pubmed/advanced) - [NOT in builder](https://www.ncbi.nlm.nih.gov/pubmed/advanced) - [Delete from history](https://www.ncbi.nlm.nih.gov/pubmed/advanced) - [Show search results](https://www.ncbi.nlm.nih.gov/pubmed/advanced) - [Show search details](https://www.ncbi.nlm.nih.gov/pubmed/advanced) - [Save in My NCBI](https://www.ncbi.nlm.nih.gov/pubmed/advanced) - [AND in builder](https://www.ncbi.nlm.nih.gov/pubmed/advanced) - [OR in builder](https://www.ncbi.nlm.nih.gov/pubmed/advanced) - [NOT in builder](https://www.ncbi.nlm.nih.gov/pubmed/advanced) - [Show search results](https://www.ncbi.nlm.nih.gov/pubmed/advanced) - [Save as a My NCBI Collection](https://www.ncbi.nlm.nih.gov/pubmed/advanced)  \| Search \| Query \| Items found \| \| --- \| --- \| --- \| \| [#6](https://www.ncbi.nlm.nih.gov/pubmed) \| Search (#2 AND #3 AND #5) \| [547](https://www.ncbi.nlm.nih.gov/pubmed/?cmd=HistorySearch&querykey=6) \| \| [#5](https://www.ncbi.nlm.nih.gov/pubmed) \| Search Return to Sport[Mesh] OR Return to Sport*[tiab] OR return to play[tiab] OR Return to Sporting Activit*[tiab] OR Resumption of Sporting Activit*[tiab] OR Sporting Activity Resumption*[tiab] OR Resumption of Recreational Activit*[tiab] OR recreational activities resumption*[tiab] OR Return to Recreational Activit*[tiab] OR timing of return[tiab] OR return to activ*[tiab] OR (time AND return) \| [39385](https://www.ncbi.nlm.nih.gov/pubmed/?cmd=HistorySearch&querykey=5) \| \| [#3](https://www.ncbi.nlm.nih.gov/pubmed) \| Search child*[tw] OR schoolchild*[tw] OR infan*[tw] OR adolescen*[tw] OR pediatri*[tw] OR paediatr*[tw] OR neonat*[tw] OR boy[tw] OR boys[tw] OR boyhood[tw] OR girl[tw] OR girls[tw] OR girlhood[tw] OR youth[tw] OR youths[tw] OR baby[tw] OR babies[tw] OR toddler*[tw] OR teen[tw] OR teens[tw] OR teenager*[tw] OR newborn*[tw] OR postneonat*[tw] OR postnat*[tw] OR perinat*[tw] OR puberty[tw] OR preschool*[tw] OR suckling*[tw] OR picu[tw] OR nicu[tw] OR "Arthritis, Juvenile"[Mesh] OR "Myoclonic Epilepsy, Juvenile"[Mesh] OR "Leukemia, Myelomonocytic, Juvenile"[Mesh] OR "Xanthogranuloma, Juvenile"[Mesh] OR "Juvenile Delinquency"[Mesh] OR "Corneal Dystrophy, Juvenile Epithelial of Meesmann"[Mesh] OR "Young Adult"[Mesh] OR young adult*[tiab] \| [4722787](https://www.ncbi.nlm.nih.gov/pubmed/?cmd=HistorySearch&querykey=3) \| \| [#2](https://www.ncbi.nlm.nih.gov/pubmed) \| Search Anterior Cruciate Ligament[Mesh] OR Anterior Cruciate Ligament Reconstruction[Mesh] OR Anterior Cruciate Ligament Injuries[Mesh] OR Anterior cruciate ligament*[tiab] OR anterior cranial cruciate ligament*[tiab] OR cranial cruciate ligament*[tiab] OR cruciate cranial ligament*[tiab] OR Bone-Patellar Tendon-Bone Grafting[tiab] OR ACL[tiab] \| [25383](https://www.ncbi.nlm.nih.gov/pubmed/?cmd=HistorySearch&querykey=2) \|  - [AND in builder](https://www.ncbi.nlm.nih.gov/pubmed) - [OR in builder](https://www.ncbi.nlm.nih.gov/pubmed) - [NOT in builder](https://www.ncbi.nlm.nih.gov/pubmed) - [Delete from history](https://www.ncbi.nlm.nih.gov/pubmed) - [Show search results](https://www.ncbi.nlm.nih.gov/pubmed) - [Show search details](https://www.ncbi.nlm.nih.gov/pubmed) - [AND in builder](https://www.ncbi.nlm.nih.gov/pubmed) - [OR in builder](https://www.ncbi.nlm.nih.gov/pubmed) - [NOT in builder](https://www.ncbi.nlm.nih.gov/pubmed) - [Delete from history](https://www.ncbi.nlm.nih.gov/pubmed) - [Show search results](https://www.ncbi.nlm.nih.gov/pubmed) - [Show search details](https://www.ncbi.nlm.nih.gov/pubmed) - [Save in My NCBI](https://www.ncbi.nlm.nih.gov/pubmed) - [AND in builder](https://www.ncbi.nlm.nih.gov/pubmed) - [OR in builder](https://www.ncbi.nlm.nih.gov/pubmed) - [NOT in builder](https://www.ncbi.nlm.nih.gov/pubmed) - [Show search results](https://www.ncbi.nlm.nih.gov/pubmed) - [Save as a My NCBI Collection](https://www.ncbi.nlm.nih.gov/pubmed) |
| Embase | Database(s): **Embase** 1974 to 2020 Week 13  Search Strategy:   \| **#** \| **Searches** \| **Results** \| \| --- \| --- \| --- \| \| 1 \| exp anterior cruciate ligament/ or exp anterior cruciate ligament reconstruction/ or exp anterior cruciate ligament injury/ or (anterior cruciate knee ligament* or anterior cruciate ligament* or anterior cranial cruciate ligament* or cranial cruciate ligament* or Bone-Patellar Tendon-Bone Grafting or ACL).ab,ti. \| 33171 \| \| 2 \| exp *adolescence/ or exp *adolescent/ or exp *child/ or exp *childhood disease/ or exp *infant disease/ or exp young adult/ or (adolescen* or babies or baby or boy? or boyfriend or boyhood or girlfriend or girlhood or child or child* or child*3 or children* or girl? or infan* or juvenil* or juvenile* or kid? or minors or minors* or neonat* or neo-nat* or newborn* or new-born* or paediatric* or peadiatric* or pediatric* or perinat* or preschool* or puber* or pubescen* or school* or teen* or toddler? or underage? or under-age? or youth* or young adult*).ab,ti. \| 3988974 \| \| 3 \| exp return to sport/ or (return to sport* or resumption to sport* or return to play* or return to sporting* or (tim* and return) or return to activ*).ab,ti. \| 54409 \| \| 4 \| 1 and 2 and 3 \| 429 \| |
