# Supplementary material for: Limited evidence for return to sport testing after ACL reconstruction in children and adolescents under 16 years: a scoping review
Source: J Exp Orthop. 2020 Oct 15;7:83. doi: 10.1186/s40634-020-00298-8 (PMC7561621; doi:10.1186/s40634-020-00298-8)
Supplement: Supplementary file 2 — Additional file 2. [file 40634_2020_298_MOESM2_ESM.docx]

# Appendix 2 – Overview of studies for each test category

Table 1. Overview of studies analysing muscle strength.

| **Muscle strength tests** | | | **Beischer [4]** | **Burland [9]** | **Clagg [11]** | **Fryer [14]** | **Hannon [16]** | **Ithurburn [18]** | **Ithurburn [19]** | **Ithurburn [21]** | **Ithurburn [22]** | **Palmieri-Sm. [29]** | **Schmitt [34]** | **Schmitt [35]** | **Toole [36]** |  |
| --- | --- | --- | --- | --- | --- | --- | --- | --- | --- | --- | --- | --- | --- | --- | --- | --- |
| **Quadriceps** | Isometric | | X | X |  | X |  |  | X | X | X |  | X | X |  |  |
|  | Isokinetic | 60⁰/s |  | X |  |  | X |  |  |  |  | X |  |  |  |  |
|  |  | 90⁰/s | X |  |  |  |  |  |  |  |  |  |  |  |  |  |
|  |  | 180⁰/s |  | X | X |  |  | X |  |  | X |  |  |  | X |  |
|  |  | 300⁰/s |  | X |  |  |  |  |  |  | X |  |  |  |  |  |
| **Hamstrings** | Isometric | | X | X |  |  |  |  |  |  |  |  |  |  |  |  |
|  | Isokinetic | 60⁰/s |  | X |  |  | X |  |  |  |  | X |  |  |  |  |
|  |  | 90⁰/s | X |  |  |  |  |  |  |  |  |  |  |  |  |  |
|  |  | 180⁰/s |  | X | X |  |  | X |  |  | X |  |  |  | X |  |
|  |  | 300⁰/s |  | X |  |  |  |  |  |  | X |  |  |  |  |  |
| **Hip abduction** | Not specified | |  |  |  |  | X |  |  |  |  |  |  |  |  |  |
|  | Isokinetic 120⁰/s | |  |  | X | X |  |  |  |  | X |  |  |  |  |  |
| **Hip external rotation** | | |  |  |  |  | X |  |  |  |  |  |  |  |  |  |
| **Study data** | | | | | | | | | | | | | | | | |
| Number of patients (for each group as defined within the study) | | | 384 (8m)  271  (12m) | 34 (RTS)  16 (not RTS) | 66 (ACLR)  47 (c) | 130 (ACLR)  56 (c) | 44 (pass)  4 (fail) | 16 (ped)  113 (adol)  15 (y.a.) | 36 (HQ)  36 (LQ) | 52 (HQ)  41 (LQ)  47 (c) | 67 | 7 ACLx1  7 ACLx2  7 (c) | 37 (HQ)  31 (LQ)  47 (c) | 55 (ACLR)  35 (c) | 88 (♀)  27 (♂) |  |
| Timing tests (months after ACLR) | | | 8-12 | 3-6 | 7  At RTS | 8  At RTS | 7  At RTS | 8.5  At RTS | 8  At RTS | 7-8  At RTS | 8  At RTS | 6-8  At RTS | 8  At RTS | 7  At RTS | 8  At RTS |  |
| As prognostic value for* | | | - | 6 | - | - | - | - | 2 & 3 | 1 | 2 | 5 | - | - | 4 |  |
| Follow-up | | | N/A | 7m | N/A | N/A | N/A | N/A | 1y | N/A | 2y | >3y | N/A | N/A | 1y |  |

ACLR = anterior cruciate ligament reconstruction; ACLx1 = single ACL injury; ACLx2 = double ACL injury; adol = adolescents; c = controls; m = months; HQ = high quadriceps; LQ = low quadriceps; N/A = not applicable; ped = pediatric; RTS = return to sport; y = years; y.a. = young adults.

* 1. Movement quality; 2. PROMs; 3. Hop tests; 4. Combined test criterion cut-offs; 5. Re-ruptures (only ipsilateral); 6. Achieving RTS.

Table 2. Overview of studies analysing hop tests.

| **Hop tests** | **Beischer [4]** | **Ithurburn [18]** | **Toole [36]** | **Wren [43]** |
| --- | --- | --- | --- | --- |
| Noyes’ hop tests battery |  | X | X |  |
| Single leg hop for maximal distance | X |  |  | X |
| Unilateral vertical hop | X |  |  |  |
| Side hop | X |  |  |  |
| **Study data** | | | | |
| Number of patients (for each group as defined within the study) | 384  (8m)  271  (12m) | 16 (ped)  113 (adol)  15 (y.a.) | 88 (♀)  27 (♂) | 29 (SYM)  17 (ASYM)  24 (c) |
| Timing tests (months after ACLR) | 8-12 | 8.5  At RTS | 8  At RTS | 7  Before RTS |
| As prognostic value for* | - | - | Test criterion cut-offs | - |
| Follow-up | N/A | N/A | 1y | N/A |

ACLR = anterior cruciate ligament reconstruction; adol = adolescent; ASYM = asymmetric; c = control; m = months; ped = pediatric; N/A = not applicable; RTS = return to sport; SYM = symmetric; y = years; y.a. = young adult.

Table 3. Overview of studies analysing movement quality.

| **Movement quality** | **Boyle [7]** | **Capin 10]** | **Clagg [11]** | **Fryer [14]** | **Hannon [16]** | **Ithurburn [20]** | **Ithurburn [21]** | **Myer [27]** | **Palmieri-Smith [29]** | **Paterno [31]** | **Paterno [32]** | **Paterno [33]** | **Schmitt [34]** | **Wren [43]** |
| --- | --- | --- | --- | --- | --- | --- | --- | --- | --- | --- | --- | --- | --- | --- |
| **Landing variables** |  |  |  |  |  |  |  |  |  |  |  |  |  |  |
| **General** |  |  |  |  |  |  |  |  |  |  |  |  |  |  |
| Peak vertical ground reaction force |  |  |  |  |  |  | X | X |  |  |  | X | X | X |
| Peak loading rate |  |  |  |  |  |  | X |  |  |  |  |  | X |  |
| **Postural** |  |  |  |  |  |  |  |  |  |  |  |  |  |  |
| Frontal Plane Trunk Excursion |  |  |  | X | X | X | X |  |  |  |  |  |  |  |
| Pelvic tilt |  |  |  |  |  |  |  |  |  |  |  |  |  | X |
| Pelvic obliquity |  |  |  |  |  |  |  |  |  |  |  |  |  | X |
| **Hip** |  |  |  |  |  |  |  |  |  |  |  |  |  |  |
| Flexion angle |  |  |  |  |  |  |  |  |  |  |  | X |  | X |
| Average flexion moment |  |  |  |  |  |  |  |  |  |  |  |  |  | X |
| Adduction angle |  |  |  |  |  |  |  |  |  |  |  | X |  | X |
| Rotation angle |  |  |  |  |  |  |  |  |  |  |  | X |  | X |
| Energy absorption |  |  |  |  |  |  |  |  |  |  |  |  |  | X |
| **Knee** |  |  |  |  |  |  |  |  |  |  |  |  |  |  |
| Valgus angle |  |  |  |  | X |  |  |  |  |  |  |  |  |  |
| Extension angle |  |  |  |  |  |  |  |  |  |  |  | X |  |  |
| Peak internal extension moment |  |  |  |  |  | X | X |  |  |  |  |  | X |  |
| Flexion angle |  |  |  |  |  |  |  |  |  |  |  |  |  | X |
| Flexion excursion |  |  |  |  | X | X | X |  |  |  |  |  | X |  |
| Peak flexion |  |  |  |  |  |  |  |  |  |  |  |  | X |  |
| Peak flexion moment |  |  |  |  |  |  |  |  |  |  |  |  | X |  |
| Average flexion moment |  |  |  |  |  |  |  |  |  |  |  |  |  | X |
| Adduction angle |  |  |  |  |  |  |  |  |  |  |  | X |  | X |
| Average adduction moment |  |  |  |  |  |  |  |  |  |  |  |  |  | X |
| Internal rotation angle |  |  |  |  |  |  |  |  |  |  |  | X |  |  |
| Energy absorption |  |  |  |  |  |  |  |  |  |  |  |  |  | X |
| **Ankle** |  |  |  |  |  |  |  |  |  |  |  |  |  |  |
| Dorsiflexion angle |  |  |  |  |  |  |  |  |  |  |  |  |  | X |
| Average dorsiflexion moment |  |  |  |  |  |  |  |  |  |  |  |  |  | X |
| Energy absorption |  |  |  |  |  |  |  |  |  |  |  |  |  | X |
| **Muscle (p)reactivity (EMG)** |  |  |  |  |  |  |  |  |  |  |  |  |  |  |
| Vastus lateralis |  |  |  |  |  |  |  |  | X |  |  |  |  |  |
| Biceps femoris |  |  |  |  |  |  |  |  | X |  |  |  |  |  |
| Lateral gastrocnemeus |  |  |  |  |  |  |  |  | X |  |  |  |  |  |
| **Gait pattern variables** |  | X |  |  |  |  |  |  |  |  |  |  |  |  |
| Peak knee flexion angle |  | X |  |  |  |  |  |  |  |  |  |  |  |  |
| Peak knee adduction angle |  | X |  |  |  |  |  |  |  |  |  |  |  |  |
| Peak internal knee extension moment |  | X |  |  |  |  |  |  |  |  |  |  |  |  |
| Peak internal knee adduction moment |  | X |  |  |  |  |  |  |  |  |  |  |  |  |
| Peak knee extensor muscle forces |  | X |  |  |  |  |  |  |  |  |  |  |  |  |
| Knee flexor muscle forces at peak internal knee extension moment |  | X |  |  |  |  |  |  |  |  |  |  |  |  |
| Peak knee flexor muscle forces |  | X |  |  |  |  |  |  |  |  |  |  |  |  |
| Peak medial compartment tibiofemoral contact forces |  | X |  |  |  |  |  |  |  |  |  |  |  |  |
| **Balance tests and postural stability** |  |  |  |  |  |  |  |  |  | X | X | X |  |  |
| Star excursion balance test, distance reached |  |  | X |  |  |  |  |  |  |  |  |  |  |  |
| Variability in ankle motion |  |  |  |  |  |  |  |  |  | X |  |  |  |  |
| Variability in hip motion |  |  |  |  |  |  |  |  |  | X |  |  |  |  |
| Postural coordination patterns between hip and ankle |  |  |  |  |  |  |  |  |  | X |  |  |  |  |
| Postural sway in degrees |  |  |  |  |  |  |  |  |  |  | X | X |  |  |
| **Other** |  |  |  |  |  |  |  |  |  |  |  |  |  |  |
| FMS | X |  |  |  |  |  |  |  |  |  |  |  |  |  |
| LQYB test | X |  |  |  |  |  |  |  |  |  |  |  |  |  |
| **Study data** | | | | | | | | | | | | | | |
| Number of patients (for each group as defined within the study) | 17 (SI)  22 (SM)  16 (adult) | 7 (ACLx1)  7 (ACLx2) | 66 (ACLR)  47 (c) | 130 (ACLR)  56 (c) | 44 (pass)  4 (fail) | 41† | 52 (HQ)  41 (LQ)  47 (c) | 33 (ACLR)  67 (c) | 7 (ACLx1)  7 (ACLx2)  7 (c) | 14 (ACLx1)  14 (ACLX2) | 56 (ACLR)  42 (c) | 43 (ACLx1)  13 (ACLx2) | 37 (HQ)  31 (LQ)  47 (c) | 29 (SYM)  17 (ASYM)  24 (c) |
| Timing tests (months after ACLR) | 9  At RTS | 5  Before RTS | 7  At RTS | 8  At RTS | 7  At RTS | 7-8  At RTS | 7-8  At RTS | 10  After RTS | 6  At RTS | 8  At RTS | 7  At RTS | ?  At RTS | 8  At RTS | 7  Before RTS |
| As prognostic value for* | - | 3 | - | - | - | 1 & 2 | - | - | 3 | 3 | - | 3 | - | - |
| Follow-up | N/A | 2y | N/A | N/A | N/A | 2y | N/A | N/A | >3y | 1y | N/A | 1y | N/A | N/A |

ACLR = ACL reconstruction; ACLx1 = single ACL injury; ACLx2 = double ACL injury; ASYM = asymmetric; c = control; EMG = electromyography; FMS = Functional Movement Competency; HQ = high quadriceps; LQ = low quadriceps; LQYB = Lower Quarter Y-balance; m = months; N/A = not applicable; RTS = return to sport; SI = skeletally immature; SM = skeletally mature; SYM = symmetric; y= years.

†divided in SYM and ASYM for each landing variable.

* 1. PROMs; 2. Hop tests; 3. Re-ruptures (only ipsilateral [29], ipsi- and contralateral [10, 31, 33])

Table 4. Overview of studies analysing patient reported outcome measures (PROMs)

| **PROMs** | **Astur [3]** | **Beischer [4]** | **Burland [9]** | **Dekker [12]** | **Ithurburn [18]** | **Ithurburn [21]** | **McPherson [23]** | **Paterno [30]** | **Toole [36]** | **Zwolski [44]** |
| --- | --- | --- | --- | --- | --- | --- | --- | --- | --- | --- |
| IKDC |  |  |  |  | X |  |  |  | X | X |
| KOOS |  |  |  |  |  | X |  |  |  |  |
| Pedi-IKDC |  |  | X |  |  |  |  |  |  |  |
| Lysholm | X |  |  |  |  |  |  |  |  |  |
| K-SES |  | X |  |  |  |  |  |  |  |  |
| ACL-RSI |  | X | X |  |  |  | X |  |  |  |
| Tegner | X | X |  |  |  |  |  |  |  |  |
| Marx Activity Scale |  |  |  | X |  |  |  |  |  |  |
| TSK |  |  |  |  |  |  |  | X |  |  |
| **Study data** | | | | | | | | | | |
| Number of patients (for each group as defined within the study) | 34 (ACLx1)  18 (ACLx2) | 384 (8m)  271  (12m) | 34 (RTS)  16 (not RTS) | 58 (ACLx1)  27 (ACLx2) | 16 (ped)  113 (adol)  15 (y.a.) | 52 (HQ)  41 (LQ)  47 (c) | 103 (ACLx1)†  29 (ACLx2)† | 19 (high fear)  21 (low fear) | 88 (♀)  27 (♂) | 68 (high IKDC)  71 (low IKDC) |
| Timing tests (months after ACLR) | 7.5  At RTS | 8-12 | 3-6 | ?  At latest follow-up | 8.5  At RTS | 7-8  At RTS | 12  At RTS | 8  At RTS | 8  At RTS | 8  At RTS |
| As prognostic value for* | 3 | - | 4 | 3 & 4 | - | 1 | 3 | 3 | 2 | - |
| Follow-up | 2y | N/A | 7m | 2y | N/A | N/A | 2-4y | 2y | 1y | N/A |

ACLR = anterior cruciate ligament reconstruction; ACL-RSI = ACL Return to Sport after Injury; ACLx1 = single ACL injury; ACLx2 = double ACL injury; adol = adolescent; c = control; HQ = high quadriceps; (Pedi-)IKDC = (Pediatric) International Knee Documentation Committee; K-SES = Knee Self Efficacy Scale; KOOS = Knee injury and Osteo-arthritis Outcome Score; LQ = low quadriceps; m = months; N/A = not applicable; ped = pediatric; RTS = return to sport; TSK = Tampa Scale for Kinesiophobia; y = years; y.a. = young adult.

† within population < 20 years of age

* 1. Movement Quality; 2. Combined test criterion cut-offs; 3. Re-ruptures (only ipsilateral [3], ipsi- and contralateral [12, 23, 30]); 4. Achieving RTS.

Table 5. Overview of studies analysing physical examination.

| **Physical examination** | **Boyle [7]** | **Hannon [16]** | **Ithurburn [22]** | **Paterno [33]** |
| --- | --- | --- | --- | --- |
| **Laxity tests** |  |  |  |  |
| KT-1000 | X |  | X | X |
| **Range of motion** |  |  |  |  |
| Knee |  | X | X |  |
| Hip |  | X |  |  |
| Ankle |  | X |  |  |
| **Other** |  |  |  |  |
| Knee joint effusion |  |  | X |  |
| **Study data** | | | | |
| Number of patients (for each group as defined within the study) | 17 (SI)  22 (SM)  16 (adult) | 44 (pass)  4 (fail) | 67 | 43 (ACLx1)  13 (ACLx2) |
| Timing tests (months after ACLR) | 9  At RTS | 7  At RTS | 8  At RTS | ?  At RTS |
| As prognostic value for* | - | - | 1 | 2 |
| Follow-up | N/A | N/A | 2y | 1y |

ACLR = anterior cruciate ligament reconstruction; ACLx1 = single ACL injury; ACLx2 = double ACL injury; KT-1000 = Knee laxity Testing device; m = months; N/A = not applicable; RTS = return to sport; SI = skeletally immature; SM = skeletally mature; y = years.

* 1. PROMs; 2. Re-ruptures (ipsi- and contralateral).

Table 6. Overview of studies analysing test batteries.

| **Test battery** | **Ithurburn [18]** | **Toole [36]** |
| --- | --- | --- |
| **Combination test criterion cut-offs** | X | X |
| Muscle strength LSI ≥90% | X | X |
| IKDC score ≥90 | X | X |
| Hop tests LSI ≥90% | X | X |
| **Study data** | | |
| Number of patients (for each group as defined within the study) | 16 (ped)  113 (adol)  15 (y.a.) | 88 (♀)  27 (♂) |
| Timing tests (months after ACLR) | 8.5  At RTS | 8  At RTS |
| As prognostic value for | - | Maintaining sport level |
| Follow-up | N/A | 1y |

ACLR = anterior cruciate ligament reconstruction; adol = adolescent; IKDC = International Knee Documentation Committee; LSI = limb symmetry index; m = months; N/A = not applicable; ped = pediatric; RTS = return to sport; y = years; y.a. = young adult.
